# Antioxidant vitamins C, E and coenzyme Q10 vs Dexamethasone: comparisons of their effects in pulmonary contusion model

**DOI:** 10.1186/1749-8090-7-92

**Published:** 2012-09-26

**Authors:** Mertol Gokce, Ozkan Saydam, Volkan Hanci, Murat Can, Burak Bahadir

**Affiliations:** 1 Department of Thoracic Surgery, Karaelmas University Medical Faculty, Zonguldak, Turkey; 2 Department of Anesthesiology and Intensive Care Medicine, Karaelmas University Medical Faculty, Zonguldak, Turkey; 3 Department of Biochemical, Karaelmas University Medical Faculty, Zonguldak, Turkey; 4 Department of Pathology, Karaelmas University Medical Faculty, Zonguldak, Turkey

## Abstract

**Background:**

The goal of our study is to evaluate the effects of antioxidant vitamins (vitamin C and E), Coenzyme Q10 (CoQ10) and dexamethasone (Dxm) in experimental rat models with pulmonary contusion (PC).

**Methods:**

Rats were randomly divided into six groups. Except for the control, all subgroups had a moderate pulmonary contusion. Animals in the group I and group II received intraperitoneal saline, group III received 10mg.kg-1 CoQ10 group IV received 100mg.kg-1 vitamin C, group V received 150mg.kg-1 vitamin E, and group VI received 10mg.kg-1 Dxm. Blood gas analysis, serum nitric oxide (NO) and malondialdehyde (MDA) levels as well as superoxide dismutase (SOD) activity assays, bronchoalveolar lavage (BAL) fluid and histopathological examination were performed.

**Results:**

Administration of CoQ10 resulted in a significant increase in PaO2 values compared with the group I (p = 0.004). Levels of plasma MDA in group II were significantly higher than those in the group I (p = 0.01). Early administration of vitamin C, CoQ10, and Dxm significantly decreased the levels of MDA (p = 0.01). Lung contusion due to blunt trauma significantly decreased SOD activities in rat lung tissue compared with group I (p = 0.01). SOD levels were significantly elevated in animals treated with CoQ10, Vitamin E, or Dxm compared with group II (p = 0.01).

**Conclusions:**

In our study, CoQ10, vitamin C, vitamin E and Dxm had a protective effect on the biochemical and histopathological outcome of PC after experimental blunt thorax trauma.

## Background

Pulmonary contusion (PC) is the most frequently diagnosed intrathoracic injury related to blunt chest trauma, affecting 17-25% of adult blunt chest trauma patients [1,2]. It is also an independent risk factor for the development of pneumonia, severe clinical acute lung injury (ALI) and acute respiratory distress syndrome (ARDS) [1]. The exact mechanisms of PC due to blunt trauma are not yet understood. It may lead to a variety of pathophysiological alterations including inflammation, increased alveolo-capillary permeability, pulmonary oedema, ventilation/perfusion mismatching, increased intrapulmonary shunting, and a loss of compliance [3-8]. PC is revealed to be related to a progressive inflammatory response mediated by local and systemic immunological alterations [8-10]. Cytokines, reactive oxygen species (ROS), and proteolytic enzymes released from macrophages and leucocytes increase the alveolo- capillary membrane permeability and microvascular leakage [11,12]. Although the standard treatment options for these patients remain largely supportive, such as supplemental oxygen, cardiopulmonary monitoring, analgesia and pulmonary hygiene, there is accumulating support for treating the activation of a secondary inflammatory response [6,8-10]. Early supportive approaches, especially for moderate to severe patients with PC in intensive care units, are very important. To understand and evaluate the inflammatory process, antioxidant-oxidant mechanisms, and the effect of the anti-inflammatory drugs after blunt thorax trauma, many experimental animal trials have been conducted [13-16].

Animal experiments and clinical trials have shown that the administration of coenzyme Q_10 (_CoQ_10_), which has membrane stabilizing properties and acts as an antioxidant in both mitochondrial and lipid membranes, can inhibit the elevation of serum creatine kinase activity and lipid peroxidation and reduce exercise-induced muscular injuries [17-20]. Certain vitamins, including vitamin C and vitamin E, are also suggested to have strong free radical scavenging properties [21,22]. The antioxidant drug Tempol®, antioxidant effectivity N-acetyl cystein (NAC) and dexamethasone (Dxm) were also used in experimental PC models [13,16].

The goal of our study was to evaluate the effects of antioxidant vitamins and CoQ_10_ in experimental rat models with PC by using isolated blunt thorax trauma and to evaluate the biochemical and histopathological effects by comparing the effects of these antioxidants with the potent anti-inflammatory agent dexamethasone.

## Methods

### Animals

After the study was approved by the Animal Ethics Committee of Zonguldak Karaelmas University (ZKU) Medical School, 42 adult male Wistar–Albino rats weighting 450-550 g were randomly divided into six groups. All animals were housed in the ZKU Experimental Animals Research Unit in climate controlled rooms (24 ± 1°C) on a 12 h light–12 h dark cycle. Food and water were available *ad libitum*. They were fed with food pellets produced specially for experimental animals. Experiments were conducted according to the guiding principles for the care and use of laboratory animals (NIH publication No. 85-23, revised 1985).

### Blunt chest trauma model

Lung contusion was induced using the model of isolated bilateral lung contusion described by Raghavendran *et al.*[14]. A hollow cylindrical weight (400 g) was dropped from a definite height (50 cm); it was encased in a vertical stainless steel tube that was positioned on a Lexon platform. This device was then suspended on Teflon guides in order to minimize friction and facilitate energy transfer. The platform was attached to a plastic protective shield that was in direct contact with the lateral aspect of the rat. This precordial shield was designed to protect the heart form contusion and thus directed the impact energy to the chest wall of the rats bilaterally. The impact energy created via this mechanism was calculated by using the equation E = mgh [E: energy; g: gravity (9.8 m/s2); h: height form the platform (50 cm); m: mass of the cylindrical weight (0.40 kg)]. The total energy transferred to the chest wall of the rats was calculated as 1.96 J.

### Experimental protocol

Animals were randomly allocated into six groups: group I (Control, n = 7), group II (Sham = Contusion, n = 7), group III (Contusion + CoQ_10_, n = 7), group IV (Contusion + vitamin C, n = 7), group V (Contusion + vitamin E, n = 7) and group VI (Contusion + Dxm, n = 7). The rats were anesthetized with an intraperitoneal injection of 100 mg.kg^-1^ ketamine HCl (50 mg.ml^-1^, Ketalar®, Turkey). Blunt chest trauma was then performed. Analgesia was provided by morphine sulphate (0.05 mg.kg^-1^) administered intraperitoneally. Following the procedure, all subgroups were transferred to their cages. Rats were checked by observing breathing, nose bleeding, respiratory movements and cardiac rhythm. After 45 min. of observation, animals in groups I and II received intraperitoneal saline, group III was given 10 mg.kg^-1^ CoQ_10_, group IV was given 100 mg.kg^-1^ vitamin C, group V was given 150 mg.kg^-1^ vitamin E, and group VI was given 10 mg.kg^-1^ Dxm.

All subgroups were observed for 6 h in their cages. Thereafter, midsternotomy was performed on animals in all subgroups. Blood samples were collected from the descending aorta in a heparinized syringe followed by analysis with a blood gas analyzer. After the left main bronchus was clamped, bronchoalveolar lavage (BAL) of the right lung was performed with 2 ml of normal saline through a tracheal cannula. This was repeated three times; in total, 2 ml of lavage fluid was obtained. At the end of the BAL, the right lung was harvested and the upper lobe was fixed in 10% formaldehyde for histopathological examination; the remaining portion of the lung was stored at -20°C until further analysis.

### Arterial blood gas measurements

Blood samples were collected from the descending aorta in a heparinized syringe during midsternotomy performed 6 h after blunt trauma. Analysis was performed by a blood gas analyzer (Cobas 221®; Roche, Mannheim, Germany) to assess and compare the effects of various drugs administered in the early period of blunt injury on pulmonary shunting.

### Biochemical assay

Biochemical parameters were studied in the blood samples; the activity of superoxide dismutase (SOD) and the levels of malondialdehyde (MDA) and nitric oxide (NO) were analyzed. High performance liquid chromatographic analysis was performed with an isocratic method using a Shimadzu HPLC system (Kyoto, Japan) and a commercial MDA kit (Immundiagnostik AG, Bensheim, Germany). Total SOD activity was determined according to the method from Sun *et al.*[23]. The principle of the method is based on the inhibition of nitroblue tetrazolium (NBT) reduction by the xanthine–xanthine oxidase system as a superoxide generator. One unit of SOD was defined as the enzyme amount causing 50% inhibition in the NBT reduction rate. Serum NO levels (nitrite + nitrate) were measured by a spectrophotometer at 545 nm (Shimadzu, Tokyo, Japan) after conversion of nitrate to nitrite by copperized cadmium granules [24]. Results are expressed μ mol/L for MDA, μmol/L for nitric oxide and U/ml for SOD.

### PMNL count in BAL

One slide for cytologic examination was prepared from each BAL sample and stained with hematoxylin-eosin. Smears were then analyzed by using an image analysis program (Leica QWINPlus v. 3.1.0). Ten microscopic fields at x100 magnification were randomly selected. The polymorphonuclear cellularity was determined as the number of cells per 25000 μm^2^.

### Histopathological examination

Lung tissue samples were fixed in 10% formalin immediately after removal, dehydrated in graded concentrations of ethanol, cleared in xylene and embedded in paraffin. At least eight tissue sections of 5-μm thickness were obtained and then stained with hematoxylin-eosin. The sections were examined under a light microscope by a pathologist in a blinded manner. All histopathological changes were documented in each lung tissue, including intra-alveolar haemorrhage, alveolar oedema, disruption and congestion and leukocyte infiltration. Alveolar oedema and congestion were scored on a scale from 0 to 3 where 0 = absence of pathology (<5% of maximum pathology), 1 = mild (<10%), 2 = moderate (15-20%), and 3 = severe (20-25%). Leukocyte infiltration was evaluated to determinate the severity of inflammation resulting from contusion. Each section was divided into 10 subsections, and leukocytic infiltration was examined in each of the subsections at a magnification of 400X with the following scale; 0: no extravascular leukocytes; 1: <10 leukocytes; 2: 10-45 leukocytes; 3: >45 leukocytes. The average of the numbers was used for comparison [25].

### Power analysis

The primary outcome of this study was the determination of the PaO_2_ values. Sample size estimation was based on the standard deviation of a similar study performed by Türüt *et al.*[13].We used the PaO_2_ values of rats in control conditions (213.57 ± 26.34) determined by the same study (13). In order to detect a 20% change in PaO_2_ with an alpha error of 0.05 and a power of 95%, it was calculated that the sample size should be at least 7 rats per group.

### Statistical analysis

Data are presented as means ± standard deviation. The Mann Whitney *U* test was used for statistical analysis. *p* < 0.05 was defined as the level of statistical significance. A commercial software package (SPSS 11.5 for Windows, SPSS Science, Chicago, IL) was used for data analysis.

## Results

Of the 42 animals subjected to blunt chest trauma, none of the rats died during the experiment. During the follow up period of contused rats, gross evaluation of the respiratory movements indicated a moderate bradypnea early after the trauma, but when the rats were transferred to the cages and supported with oxygen, respiratory rates reflected similar values compared with the control group. All rats exhibited similar behavior concerning mobilization and activity. Median sternotomy grossly indicated the effect of blunt trauma on the lungs; both lungs showed contused areas with a heterogeneous pattern.

Arterial blood gas analysis was assessed at 6 hours after blunt trauma. Results are shown in Table 1. All PaO_2_ values decreased significantly in group II as compared with the group I (*p < 0.05*). Administration of vitamin C, vitamin E and Dxm resulted in a slight increase in PaO_2_ values compared with the group II, but the difference was not statistically significant (*p > 0.05*). Administration of CoQ_10_ resulted in a significant increase in PaO_2_ values compared with group II (*p = 0.004*). PaCO_2_ levels significantly increased in group II compared with the group I (*p = 0.01*). Compared to group II, PaCO_2_ levels were significantly decreased in the group III, group IV, group V, and group VI (*p < 0.05*). All pH values significantly decreased in group II compared with the group I (*p = 0.01*).

**Table 1:** Comparison of blood gas analysis results obtained 6 h after contusion between the control, sham and drug-administered groups

**Groups**	**pH**	**pO2**	**pCO2**
GroupI: Control **(n = 7)**	7.36 ± 0.03^b^	223.16 ± 11.67^b^	38.84 ± 2.77^b^
GroupII: Sham(Cont) **(n = 7)**	7.26 ± 0.02^a^	87.2750 ± 9.01^a^	57.48 ± 1.07^a^
GroupIII: Cont + CoQ_10_**(n = 7)**	7.30 ± 0.02^ab^	106.26 ± 8.92^ab^	48.96 ± 4.05^ab^
GroupIV: Cont + vit C **(n = 7)**	7.31 ± 0.04^ab^	100.38 ± 6.92^a^	47.44 ± 5.77^ab^
GroupV: Cont + vit E **(n = 7)**	7.30 ± 0.02^ab^	99.98 ± 5.57^a^	47.88 ± 3.57^ab^
GroupVI: Cont + Dxm **(n = 7)**	7.30 ± 0.03^ab^	102.60 ± 7.09^a^	49.80 ± 1.92^ab^

Datas are presented as mean ± S.D.

*Cont*: contusion, *Dxm*: Dexamethasone, *vit C*: vitamin C, *vit E*: vitamin E, *CoQ*_*10*_: Co enzyme Q_10_.

^a^*p < 0.05* in comparison with control.

^b^*p < 0.05* in comparison sham animals.

pH values of arterial blood gases in all groups were higher than the group II. pH values in the group IV (*p = 0.03*), and group III (*p = 0.03*) were significantly higher than the group II. However pH values of group III, group V and group VI were significantly low compared with the group I (*p < 0.05*).

### Biochemical results

Levels of plasma MDA (Table 2) in group II were significantly higher than those in the group I reflecting the significant lipid peroxidation that occurred with lung contusion after blunt trauma (*p = 0.01*). Early administration of vitamin C, CoQ_10_, and Dxm significantly decreased the levels of MDA (*p = 0.01*). No significant differences in MDA values were found between the group V and group II (*p = 0.06*).

**Table 2:** The activites of MDA, SOD and NO in serum

**Groups**	**MDA (μmol/mg prot)**	**SOD (U/mg prot)**	**NO (μmol/L)**
GroupI: Control **(n = 7)**	1.41 ± 0.16^b^	4.38 ± 0.52^b^	38.59 ± 3.55^b^
GroupII: Sham(Cont) **(n = 7)**	2.68 ± 0.37^a^	1.14 ± 0.49^a^	82.61 ± 8.01^a^
GroupIII: Cont + CoQ_10_**(n = 7)**	1.67 ± 0.39^b^	2.86 ± 0.53^ab^	51.30 ± 5.89^ab^
GroupIV: Cont + vit C **(n = 7)**	1.71 ± 0.51^b^	**2.48 ± 0.68**^**a**^	56.36 ± 5.75^ab^
GroupV: Cont + vit E **(n = 7)**	**1.89 ± 0.40**	2.53 ± 0.34^ab^	57.61 ± 2. 30^ab^
GroupVI: Cont + Dxm **(n = 7)**	1.77 ± 0.22^b^	3.49 ± 0.25^ab^	50.98 ± 8.53^ab^

Datas are presented as mean ± S.D.

*Cont*: contusion, *Dxm*: Dexamethasone, *vit C*: vitamin C, *vit E*: vitamin E, *CoQ*_*10*_: Co enzyme Q_10_.

^a^*p < 0.05* in comparison with control.

^b^*p < 0.05* in comparison sham animals.

In group II, lung contusion due to blunt trauma, significantly decreased SOD activity in rat lung tissue compared with group I (*p = 0.01*). SOD levels were significantly elevated in the group III, group V, and group VI compared with group II (*p = 0.01*). No significant difference in SOD values was found between the group IV and group II (*p = 0.06*). In group II, the level of NO was higher than in the group I (*p = 0.01*). Early treatment with vitamin C, vitamin E, CoQ_10_ and Dxm decreased the NO levels significantly (*p = 0.01*).

### Neutrophil count in BAL

**Figure 1:**
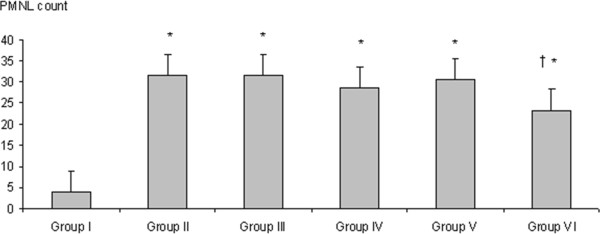
**Comparison of BAL fluid neutrophil counts per mm3 in each group.** Contusion resulted in a significant increase in neutrophil population (p = 0.003). Contusion-induced neutrophil increase was reduced significantly by Dxm. administration compared to sham group (p = 0.016). *: *p < 0.01* compared Group I, Mann whitney *U* test. †: *p < 0.05* compared Group II, Mann whitney *U* test. Group I: control. Group II: contusion (sham). Group III: contusion + CoQ_10_. Group IV: contusion + vitamin C. Group V: contusion + vitamin E. Group VI: contusion + dexamethasone.

Contusion resulted in a significant increase in the neutrophil population (*p = 0.003*). Dxm decreased the neutrophil population significantly in the BAL fluid of contused rats (*p = 0.016*) (Figure 1).

### Histopathological results

Scores of alveolar haemorrhage, oedema and disruption as well as leukocyte infiltration scores were significantly higher in group II compared to the group I (*p = 0.001*) (Table 3). In group III, CoQ_10_ administration, significantly improved the hemorrhage and oedema scores (*p = 0.011*) and the leukocyte infiltration scores (*p = 0.002*) compared to group II. There were no significant difference in the haemorrhage (*p = 0.383)* and leukocyte infiltration (*p = 0.073*) scores between the group II and group IV. There was no significant difference in the haemorrhage scores (*p = 0.209*), but there was a difference in the leukocyte infiltration scores (*p = 0.004*) between the group II and the group V. In group VI, Dxm administration significantly improved the haemorrhage and oedema scores (*p = 0.017*) as well as the leukocyte infiltration scores (*p = 0.002*) compared to the group II.

**Table 3:** Comparison of leukocyte infiltration and alveolar oedema/congestion scores of the lung tissue in each group

**Groups**	**Haemorrhage/oedema/alveolar disruption scores**	**Leukocyte infiltration**
GroupI: Control **(n=7)**	0.00±0.00	0.14±0.37
GroupII: Sham(Cont) **(n=7)**	2.71±0.48^a^	2.85±0.37^a^
GroupIII: Cont + CoQ_10_**(n=7)**	1.71±0.48^ab^	1.71±0.48^ab^
GroupIV: Cont + vit C **(n=7)**	2.42±0.53^acd^	2.28±0.48^acd^
GroupV: Cont + vit E **(n=7)**	2.28±0.48^a^	2.00±0.00^ab^
GroupVI: Cont +Dxm **(n=7)**	1.85±0.37^ab^	1.57±0.53^ab^

Datas are presented as mean ±S.D.

*Cont*: contusion, *DXM*: Dexamethasone, *vit. C*: vitamin C, *vit. E*: vitamin E, *CoQ*_*10*_: Co enzyme Q_10_.

^a^*p<0.05* in comparison with control.

^b^*p<0.05* in comparison sham animals.

^c^*p<0.05* in comparison vit C & CoQ10.

^d^*p<0.05* in comparison Dxm & vit C.

When the drug-administered groups were compared, no significant differences were found in haemorrhage and leucocyte infiltration scores (*p > 0.05*).

**Figure 2:**
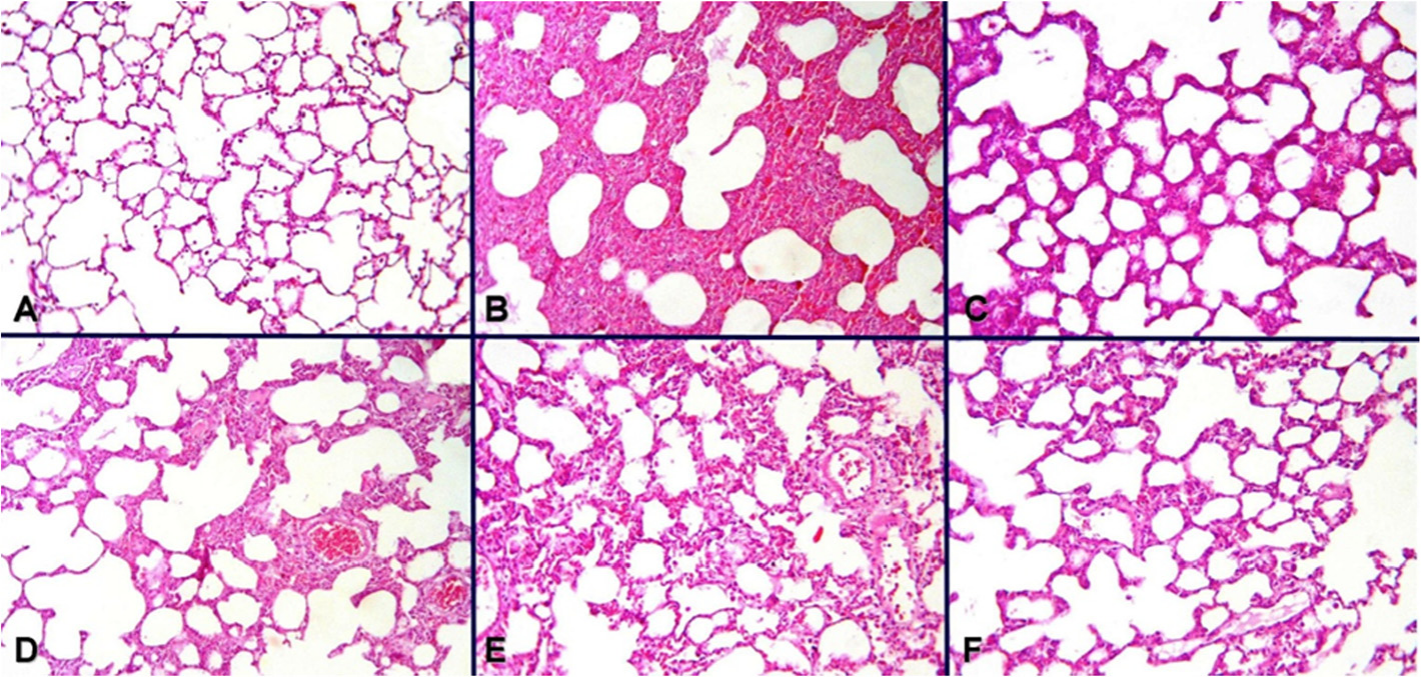
**Microphotographs demonstrating (A) control group with normal pulmonary histology; (B) sham group displaying disruption of alveolar structure with extensive congestion, oedema, and hemorrhage; and decreased congestion, and oedema with relative preservation of alveolar histology in the treatment groups: (C) vitamin C, (D) Vitamin E (E), (E) Dxm and (F) CoQ**_**10**_**.** Best improvement is appreciated in Dxm and CoQ_10_ group.

Microscopic findings in the lung specimens revealed normal lung parenchyma in the group I (Figure 2A). In contrast, rats in the group II had disruption of normal alveolar structure with severe congestion and haemorrhage associated with infiltrating leukocytes (Figure 2B). In the group IV and group V, the lungs of the contused rats exhibited moderate degrees of oedema and congestion (Figure 2C and 2D). Rats in the group VI and group III had significantly less leukocyte infiltration with relatively well- preserved alveolar histology compared to the group II (Figure 2E and 2F).

## Discussion

PC is the most frequently diagnosed injury due to blunt thorax trauma. Because approximately 1/3 of trauma patients admitted to hospitals have thorax trauma, it is an important cause of mortality and morbidity. About 25% of the patients require invasive mechanical ventilation [2,4,5,7,16]. With the development of possible new pharmacological agents and better resuscitation methods, the need for mechanical ventilation may be decreased and the prognosis of patients with PC may improve. For studying PC, various blunt thorax trauma models have been previously established. The local and systemic pathophysiological consequences of PC and the different treatment strategies to minimize the effects of this type of injury have been studied [8,10-13,26-29].

In our study, we used the rat model of lung contusion injury caused by external blunt thoracic trauma as described by Raghavendran *et al.*[14]. We generated and adapted the rat model mentioned above in order to constitute a moderate PC.

The effects of the PC depend on the extent of the injury. Direct injury causes pulmonary vascular damage with secondary alveolar haemorrhage, and thus the alveoli are poorly perfused. Afterwards, tissue inflammation develops and results in surrounding pulmonary oedema that leads to regional alterations in compliance and airway resistance causing V/Q mismatch. This manifests as a progressive deterioration of pulmonary gas exchange and P/F ratios [7]. Similar previous studies have been conducted to assess the severity of pulmonary shunting and arterial oxygenation in injured rats, revealing severe hypoxia early after induction of contusion. Although it was observed that arterial oxygenation was improved over time, levels persisted at the range of acute lung injury for 4-24 h following contusion [13,30]. In our study, all PaO_2_ levels were decreased, and all PaCO_2_ levels were increased significantly at 6 h after contusion. We detected slight improvement in both PaO_2_ and PaCO_2_ levels due to the early administration of antioxidant vitamins (vitamin C, vitamin E), CoQ_10_ and Dxm. PC is associated with a leukocyte-mediated secondary inflammatory response leading to capillary leaking, interstitial oedema and protein extravasation [5,7,10,12]. Strohmaier *et al.*[11] clearly demonstrated the importance of neutrophil influx in mediating lung injury. Alveolar levels of pro-inflammatory mediators like interleukins and TNF have been shown to rise precipitously after blunt lung injury [16]. It has been elucidated that PC is associated with a progressive inflammatory response mediated by local and systemic immunological alterations [8-13]. Macrophages and neutrophils, which are potentially involved in the inflammatory process, are activated after blunt trauma. Cytokines, reactive oxygen metabolites, and proteolytic enzymes are released by both leukocytes and macrophages.

This leads to increased alveolocapillary membrane permeability and microvascular leakage associated with the formation of alveolar oedema fluid, proteolytic and lipolytic enzymes, and reactive oxygens species (ROS) [11]. Alveolar macrophages can produce potent ROS, such as superoxide radicals and, consequently, peroxynitrite. Peroxynitrite can be produced by the reaction of NO with superoxide radicals and represents a highly oxidative species [30]. These ROS in turn are capable of initiating and promoting oxidative damage in the form of lipid peroxidation [18]. Some organs might prevent the damaging effects of the oxidant species through enzymatic and non-enzymatic antioxidant defenses. These defenses include enzymes, such as superoxide dismutase (SOD) and catalase [19]. Malondialdehyde (MDA) is a good indicator of free radical formation, and its elevation shows increased lipid peroxidation due to the effects of these radicals [25]. In our study, MDA levels showed significant increases after contusion, reflecting an indirect finding of oxidative damage. Similarly, NO levels were significantly elevated in the lung tissue. SOD activities were demonstrated to be decreased in the contused rats. These results suggest that oxidants and antioxidants participate in the mechanism of lung injury induced by PC.

The deleterious effects of the free radicals are determined by a delicate balance between the rate of their production and elimination by the different antioxidant systems. Beneficial effects of antioxidant molecules in experimental ALI, blunt trauma and PC models have been established in previous studies. [13,16,31-34]. Maxwell *et al.*[16] established the positive effects of the antioxidant drug Tempol® in a pig model with unilateral PC. Davis *et al.*[10] showed that systemic levels of prostacyclin and tromboxane elevate after PC. They also showed attenuation of pulmonary failure by pretreatment with indomethacin due to the effect of prostanoid inhibition. Koksel *et al.*[31] caused ALI in rats by intravenous administration of oleic acid and showed the positive effects of NAC, which has antioxidant properties. Türüt *et al.*[13] investigated the biochemical and histopathologic effects of dexamethasone, NAC and aprotinin in PC rat models. They reported that Dxm, NAC and aprotinin treatment have positive effects in bilateral PC, which activates an oxidant-antioxidant cascade.

CoQ10, a component of the mitochondrial electron transport chain, is a strong antioxidant that plays a role in membrane stabilization. Antioxidants inhibit lipid peroxidation by preventing a peroxidation chain reaction and/or picking up reactive oxygen derivatives. Endogenous antioxidants include mitochondrial cytochrome oxidase, SOD, catalase, glutathione peroxidase (GSH-Px), glutathione S-transferase, hydroperoxidase and CoQ. CoQ10 is the only antioxidant that is fat-soluble, synthesized endogenously and is present in tissues in active form (reduced) independently from diet [35]. It has been found in experimental studies that CoQ10 is effective in the treatment of ischemia reperfusion injury. Portakal *et al.*[36] found that CoQ10 has beneficial effects in the rat liver ischemia-reperfusion damage. Similary, Ostrowski *et al.*[37] suggested that CoQ_10_ has beneficial effects in cerebral ischemia. They also reported that CoQ_10_ reduced the cerebral ischemic lesion diameter in an animal model. Nonetheless, the effectiveness of CoQ_10_ in a trauma model has not been thoroughly studied. Kerimoglu *et al.*[38] has demonstrated that CoQ10 administration was beneficial after spinal cord trauma. Tran et al. [39] showed that pretreatment with Tempol® or CoQ10 decreased superoxide production, reduced the infarct size and normalized mitochondrial dysfunction in the gastrocnemius muscle after tourniquet-induced acute ischemia- reperfusion injury in mouse skeletal muscles. However, CoQ_10_ administration after lung injury in animal models has very rarely been studied. Lim *et al.*[40] demonstrated that CoQ_10_ reduced the increase in tumor necrosis factor-alpha (TNFα) and the injury-induced rise in peak airway pressure following lung injury models in rats. They concluded that CoQ_10_ may have a protective role in lung injury. They demonstrated that a high-dose of oral CoQ_10_ was well incorporated into plasma and lung tissue and, to a lesser extent, into the heart. Having shown that oral administration of CoQ_10_ provided substantial increases in plasma and lung tissue concentrations, the authors tested the benefits of these increases using an in situ lung injury model that incorporated physiological and biochemical measurements used by transplant clinicians to assess the function of the donor lung. The lung ischemia-reperfusion injury (IRI) model demonstrated both increased peak airway pressure, poor gas exchange and increased levels of TNFα. In this novel study of the effects of CoQ_10_ pre-dosing on lung IRI, there is evidence that oral administration of CoQ_10_ significantly attenuated the rise in cytokine TNFα but not IL-6, MDA and ATP. However, it did not provide sufficient clinical protection against IRI as judged by peak airway pressure and oxygen diffusion gradients [40]. However, there is no study evaluating the role of CoQ_10_ in a blunt chest trauma model. In our study, comparisons of MDA, SOD and NO levels measured after early administration of CoQ_10_ in experimental rat models were statistically significant. The serum MDA levels of the group that received CoQ_10_ were closest to the group I. These findings are similar to the previously published data studying the antioxidant properties of CoQ_10_[35-40]. Vitamin C is capable of scavenging oxygen-derived free radicals that are involved in the development or exacerbation of various diseases, including cancer, heart attack, arthritis and stroke [33]. Omeroglu *et al.*[34] administrated high doses of vitamin C to rat models with ruptured Achilles tendons and showed that a high dose vitamin C supplementation has positive effects on tendon healing by promoting early angiogenesis and increased collagen synthesis. Cristante *et al.*[41] demonstrated that vitamin C reduces the inflammatory response after spinal cord injury in rats. Additionally, Liao *et al.*[42] showed that early administration of vitamin C is effective for treating spinal cord injury in rats.

In our study, differences in MDA and NO levels after early administration of vitamin C in rats with blunt thorax trauma were found to be statistically significant compared to the group II. This result is consistent with previous studies demonstrating the antioxidant properties of vitamin C in PC [33,34,41,42].

Vitamin E is the major lipophilic radical-scavenging antioxidant in vivo and protects humans from the oxidative stress mediated by active oxygen and nitrogen species [43].

Koc *et al.*[44] showed that vitamin E significantly decreased MDA levels, and thus, vitamin E may have a protective effect against spinal cord injury in rats due to its antioxidant properties. In a study comparing the effect of methylprednisolone with or without vitamin E in a rat model of spinal cord damage, the size of the ischemic area was smaller, and adrenaline, noradrenaline, and dopamine levels were lower in the group treated with methylprednisolone and vitamin E [45]. Cristante *et al.*[41] demonstrated that vitamin E reduces the inflammatory response after spinal cord injury in rats. Wigenstam *et al.*[46] has also demonstrated that vitamin E reduces acute inflammatory cell influx, suppresses collagen formation in lung tissue and protects against chemically induced lung injury.

In our study, SOD and NO levels in rats who received vitamin E were statistically significant compared to the group II. This result is concordant with the previously published data describing the antioxidant properties of vitamin E [41-46]. Dexamethasone (Dxm) was also used in experimental PC models [13,16]. Türüt *et al.*[13] examined the biochemical and histopathologic effects of Dxm in a PC rat model and reported that Dxm has positive effects in bilateral PC through activation of an oxidant-antioxidant cascade.

In our study, MDA, SOD and NO levels in rats who received Dxm were statistically significant compared to the group II. The measured SOD and NO levels are the closest to the group I in our study.

Even though the beneficial effects of corticosteroids have been shown, these drugs have severe adverse effects, such as an increased risk of infection, fluid retention, increased blood pressure, high blood sugar, suppressed adrenal gland hormone production, weight gain, and loss of calcium from bones [47].

However, suitable doses and effective dose ranges of CoQ_10_ have not yet been identified. According to previous studies, 10 mg/kg CoQ_10_ was commonly administrated within one hour after injury in order to prevent ischemic damage [48]. In our study, we administrated 10 mg/kg CoQ_10_ to rat models immediately after blunt thorax trauma.

Histopathologic examination of lung tissues also supported the potential beneficial effects of the antioxidant vitamins C and E as well as Dxm and CoQ_10_. Parenchymal changes in the contused lungs were characterized by extensive haemorrhage with disruption of the alveoli, congestion, and alveolar oedema as well as increased leukocytic infiltration in the alveolar space. Although not statistically significant, vitamin C and vitamin E resulted in decreased haemorrhage, oedema and alveolar disruption. In addition, vitamin E significantly decreased leukocytic infiltration. On the other hand, administration of Dxm and CoQ_10_ subsequent to pulmonary contusion provided significant recovery. The most significant effects on haemorrhage, oedema and alveolar disruption were obtained with CoQ_10_, and dexamethasone resulted in the greatest decreases in leukocyte infiltration both in pulmonary tissue and BAL fluid. These results emphasize the antioxidant potential of the former and the anti-inflammatory property of the latter.

Finally, it appears that free oxygen radicals and lipid peroxidation play a role in PC after blunt thorax trauma. In our study, CoQ_10_, vitamin C, vitamin E and Dxm had protective effects on biochemical and histopathological outcomes of PC after experimental blunt thorax trauma. In particular, we have demonstrated that the best outcome was recorded in rats which were administered CoQ_10_ with respect to arterial oxygenation, MDA levels and histopathological outcome. Vitamin C and E are reliable and easy to administer and have been shown to have significant antioxidant effect. These results support the idea that early treatment with CoQ_10_, vitamin C, vitamin E, and dexamethasone may yield favorable effects on pulmonary pathophysiologic parameters of blunt thorax trauma patients.

## Competing interests

The author(s) (Gokce M; Saydam O, Hanci V, Can M, Bahadir B) declare that they have no competing interests.

## Authors’ contributions

Gokce M and Hanci V designed the research; Gokce M, Saydam O, Can M and Bahadir B performed the research; Hanci V, Can M and Bahadir B analyzed the data; Gokce M, Saydam O and Hanci V wrote the paper. All authors read and approved the final manuscript.
